# Dopamine prevents lipid peroxidation-induced accumulation of toxic α-synuclein oligomers by preserving autophagy-lysosomal function

**DOI:** 10.3389/fncel.2013.00081

**Published:** 2013-05-31

**Authors:** Peizhou Jiang, Ming Gan, Shu-Hui C. Yen

**Affiliations:** Department of Neuroscience, Mayo Clinic College of MedicineJacksonville, FL, USA

**Keywords:** Parkinson’s disease, α-synuclein, dopamine, autophagy, aggregation

## Abstract

The formation of Lewy bodies containing α-synuclein (α-syn), prominent loss of dopaminergic neurons and dopamine (DA) deficiency in substantia nigra and striatum are histopathological and biochemical hallmarks of Parkinson’s disease (PD). Multiple lines of evidence have indicated that a critical pathogenic factor causing PD is enhanced production of reactive oxygen species (ROS), which reacts readily with polyunsaturated fatty acids to cause lipid peroxidation (LPO). LPO products have been shown to facilitate assembly of toxic α-syn oligomers in *in vitro* studies. Since DA is prone to autoxidation and cause ROS, it has been suggested that interactions among DA, LPO, and α-syn play an important role in neuronal loss in PD. However, the exact mechanism(s) remains unclear. We addressed this issue using a neuronal cell model which inducibly expresses human wild-type α-syn by the tetracycline off (Tet-Off) mechanism and stably expresses high levels of DA transporter. Under retinoic acid elicited neuronal differentiation, cells with or without overexpressing α-syn and with or without exposure to LPO inducer-arachidonic acid (AA), plus 0–500 μM of DA were assessed for the levels of LPO, α-syn accumulation, cell viability, and autophagy. AA exposure elicited similar LPO levels in cells with and without α-syn overexpression, but significantly enhanced the accumulation of α-syn oligomers and monomers only in cultures with Tet-Off induction and decreased cell survival in a LPO-dependent manner. Surprisingly, DA at low concentrations (<50 μM) protected cells from AA cytotoxicity and α-syn accumulation. Such effects were attributed to the ability of DA to preserve autophagic-lysosomal function compromised by the AA exposure. At high concentrations (>100 μM), DA exposure enhanced the toxic effects of AA. To our knowledge, this is the first report showing biphasic effects of DA on neuronal survival and α-syn accumulation.

## INTRODUCTION

Parkinson’s disease (PD) is characterized by accumulation of neuronal inclusions containing α-synuclein (α-syn), degeneration of dopaminergic neurons and deficiency of dopamine (DA) in substantia nigra and striatum (Dauer and Przedborski, 2003). Multiple lines of evidence have indicated that enhanced production of reactive oxygen species (ROS) is a critical pathogenic factor causing PD and that ROS can be generated via autoxidation of DA (Jenner, 1991; Fahn and Cohen, 1992; Lotharius and Brundin, 2002; Tieu et al., 2003). ROS react readily with polyunsaturated fatty acids (PUFAs), which are enriched in brain phospholipids (Rapoport, 2008; Whelan, 2008), to generate peroxyl radical and cause lipid peroxidation (LPO) via propagation and chain reaction (Young and McEneny, 2001; Farooqui and Farooqui, 2011). Such changes were shown to affect membrane properties and result in neuronal dysfunction (Fernstrom, 1999; Farooqui and Farooqui, 2011). It has been reported that presence of LPO products such as 4-hydroxynonenal, 4-oxo-2-nonenal or oxidized DA can facilitate *in vitro* assembly of α-syn oligomers, but not that of α-syn filaments (Li et al., 2004; Cappai et al., 2005; Qin et al., 2007; Nasstrom et al., 2009). There are also evidences suggesting a role of soluble oligomeric α-syn species in cytotoxicity (Winner et al., 2011). These aforementioned findings along with information derived from many other studies (Li et al., 1995; Hattoria et al., 2009; Ruiperez et al., 2010) highlight a likely role of DA and LPO in PD pathogenesis, hence it is important to investigate this issue further.

In present study, we used a neuronal cell model of PD to investigate interplays among DA, LPO, α-syn assembly and cell survival. The model, referred to as 3D5/DAT, was generated from stable transfection of 3D5 cells with human DA transporter (DAT) genes to increase DA uptake. Cells of 3D5 are derived from a human neuroblastoma cell line inducible to express human wild-type α-syn via the tetracycline off (Tet-Off) mechanism (Takahashi et al., 2007). While the expression of α-syn is inducible, expression of hDAT in 3D5/DAT cells is not. It has previously been demonstrated that 3D5 cells upon Tet-Off induction and neuronal differentiation via retinoic acid (RA) treatment are capable to accumulate small amounts of α-syn oligomers and this process has very little impacts on cell viability (Ko et al., 2008; Jiang et al., 2010). In current cell-based studies, we focused on examining cultures with and without (i) overexpressing α-syn, (ii) exposure to a LPO inducer arachidonic acid (AA), which is a major PUFA in the brain and (iii) treatment with DA. These cells were assessed as to the extent of LPO, α-syn accumulation, autophagy, and cell viability.

We found that induced α-syn expression had no effect on the level of malondialdehyde (MDA), a product of LPO. However, AA treatment affected MDA levels in a time-dependent manner, regardless whether there was α-syn overexpression or not. Exposure of 3D5/DAT cells with induced α-syn to AA caused accumulation of α-syn oligomers and decrease of cell survival when compared to non-induced controls. These changes were accompanied with alteration of autophagy marker levels and that of lysosomal membrane permeability. Such impacts could be significantly reduced by co-treatment of cultures with DA at physiological concentration or rapamycin (Rapa) which is an autophagy inducer. Importantly, the co-treatment prevented cells from changes of lysosomal membrane permeability. Treatment of cells to DA at higher concentrations led to cell death and enhanced α-syn oligomer accumulation. To our knowledge, this is the first report showing biphasic effects of DA on neuronal cells.

## MATERIALS AND METHODS

### REAGENTS

Arachidonic acid, bovine serum albumin (BSA), DA, Rapa, wortmannin (WM), Trolox (TX), and Triacsin C (TC) were purchased from Sigma. LysoTracker^®^ Green DND-26 was from Invitrogen. BSA stock solution was prepared in distilled water and stored in 4^o^C. DA solution was prepared in distilled water freshly. All other reagents were prepared in dimethyl sulfoxide (DMSO) for stock solution and stored at -20^o^C according to product instruction. For treatment of cultures, the final concentrations of AA, Rapa, WM, TX, and TC in culture media are 250 μM, 20 nM, 150 nM, 100 μM, and 10 μM, respectively, and that of DA are 25–500 μM.

### GENERATION OF A CELL MODEL WITH INDUCIBLE α-syn EXPRESSION AND NON-INDUCIBLE EXPRESSION OF DOPAMINE TRANSPORTER

The cell model, referred to as 3D5/DAT, was generated via transfection of 3D5 cells with pcDNA6-hDAT or empty vector using Tfx-20 Reagent (Promega, Madison, WI, USA) and selected with blasticidin (10 μg/mL) for 4 weeks. Cells of 3D5 were derived from a human neuroblastoma BE2-M17D derived cell line and have been characterized in previous studies (Ko et al., 2008; Jiang et al., 2010). They are inducible to express human wild-type α-syn by the Tet-Off mechanism in a time-dependent manner, and responsive to RA treatment to display neuronal phenotypes (Ko et al., 2008).

### CELL CULTURES

Cultures of 3D5/DAT were maintained in Dulbecco’s Modified Eagle Medium (DMEM)/10% fetal bovine serum with 2 μg/mL Tet at 37°C and 5% CO_2_. Those intended for biochemical analysis were seeded at 1.5 × 10^5^ cells/well in 6-well plates. For spectrophotometric assay 3D5/DAT cells were seeded at 2 × 10^4^ cells/well in 24-well plates or 1 × 10^4^ in 48-well plates (Bellco Glass Inc, Vineland, NJ, USA), respectively. For cell differentiation and α-syn induction, media were replaced next day with Tet deprived Neurobasal medium (Invitrogen, Carlsbad, CA, USA), 2% B-27 supplement (antioxidant free, Invitrogen), 2 mM L-glutamine (Sigma) and 20 μM RA (Sigma-Aldrich, St Louis, MO, USA). For those without α-syn induction, medium was still supplemented with Tet. Cultures were subjected to different treatments for durations to be described later.

### WESTERN BLOT ANALYSIS

Cell cultures were harvested and centrifuged at 200 × *g* for 15 min. They were lysed in 2-(*N*-morpholino)ethanesulfonic acid (MES) buffer (Jiang et al., 2010) supplemented with phosphatase inhibitors. The cell lysates were mixed with Tricine–sodium dodecyl sulfate (SDS) sample buffer (Invitrogen) and 2% β-mercaptoethanol, boiled for 5 min and resolved by SDS-polyacrylamide gel electrophoresis (SDS-PAGE) using 10–20% Tris/Tricine gels (Bio-Rad, Hercules, CA, USA). Precision plus protein standards (Bio-Rad) were included as references. Proteins separated by SDS-PAGE were transferred onto nitrocellulose paper and processed for immunolabeling for proteins of interest. Membranes were incubated with antibodies against: DAT (PRB-330P, Covance), α-syn (Synuclein-1, BD Biosciences), LC3 (Novus Biologicals), cleaved Caspase 3 (Cell Signaling), total mammalian target of rapamycin (mTOR; Cell Signaling), Ser2448 phosphorylated mTOR (Cell Signaling), and glyceraldehyde 3-phosphate dehydrogenase (GAPDH). Immunoreactivity was visualized with enhanced chemiluminescence (ECL plus, Amersham Pharmacia Biotech, Buckinghamshire, UK) or SuperSignal West Femto Maximum Sensitivity Substrate (Thermo Scientific, Rockford, IL, USA) and analyzed as before (Jiang et al., 2010).

### LIPID PEROXIDATION ASSAY

The extent of LPO was determined using Lipid Peroxidation Microplate Assay Kit (Oxford Biomedical Research), which assesses the level of MDA derived from PUFA peroxides. Briefly, cells were thoroughly washed in ice-cold 20 mM phosphate-buffered saline (PBS; pH 7.4), resuspended in PBS to obtain a density of 5 × 10^7^ cells per mL and followed by sonication in the presence of 10 μL 0.5 M butylated hydroxytoluene per 1 mL of cell suspension. Cell lysates were centrifuged at 3,000 × *g* and 4°C for 10 min to remove large particles and then quantified for protein concentration with Bicinchoninic acid Protein Assay Kit (Pierce). For LPO determination, 140 μL of standards or samples, 455 μL of diluted Reagent R1 and 105 μL 37% hydrochloric acid (HCl) were added into a microcentrifuge tube sequentially, mixed and incubated at 45°C for 60 min. The mixtures were then centrifuged at 15,000 × *g* for 10 min to obtain a clear supernatant. The supernatants were added into a microplate with a triplicate of 150 μL per well for each sample and read at 586 nm using Spectra Max M4.

### CELL VIABILITY

Cell viability was assessed using Calcein AM (Invitrogen). Cultures of 3D5/DAT cells grown in 24- or 48-well plates were incubated with 2 μM Calcein AM in balanced salt solution for 30 min at room temperature in the dark. Fluorescence signals emitted from live cells by esterase-metabolized calcein were measured at 495 nm (excitation)/530 nm (emission) using a Spectra Max M4 and Soft Max Protein 4.6 software (Molecular Devices, Sunnyvale, CA, USA). All measurements were performed in triplicate from three experiments.

### IMMUNOCYTOCHEMISTRY

Cells grown on coverslips were rinsed with PBS, fixed in 4% paraformaldehyde (PFA) and permeabilized with 0.1 M Tris-buffered saline (TBS, pH 7.6) containing 0.5% triton X-100 for 5 min. They were subsequently blocked with 3% goat serum in TBS, incubated with antibody Synuclein-1 and DAT in TBS containing 1% goat serum overnight at 4^o^C then incubated for 1 h with secondary antibodies of goat anti-mouse conjugated with Alexa594 and goat anti-rabbit antibody conjugated with Alexa488. Immunolabeled cells were stained with nuclei stain 4′,6-diamidino-2-phenylindole (DAPI; Invitrogen) for 10 min and evaluated by confocal fluorescence microscopy (Zeiss LSM 510, Carl Zeiss MicroImaging).

### LYSOTRACKER LABELING OF LYSOSOMES

Differentiated cells with α-syn induction were cultured in Lab-Tek Chambered Coverglass and subjected to different treatments for certain time. At the end of treatment, LysoTracker Green DND-26 was added at a final concentration of 100 nM to each well for 1 h. Cells were then photographed by confocal microscopy. Fluorescence intensities from images in different groups were statistically analyzed for evaluation of lysosomal function using Zen software.

### STATISTICAL ANALYSIS

Data from at least three sets of independent experiments were analyzed by one-way Anova with Dunnett’s *post hoc* test and Student’s *t*-test for comparison of groups ≥3 and 2, respectively, for statistical significance.

## RESULTS

### EXPRESSION OF DOPAMINE TRANSPORTER IN 3D5/DAT CELLS

**FIGURE 1:**
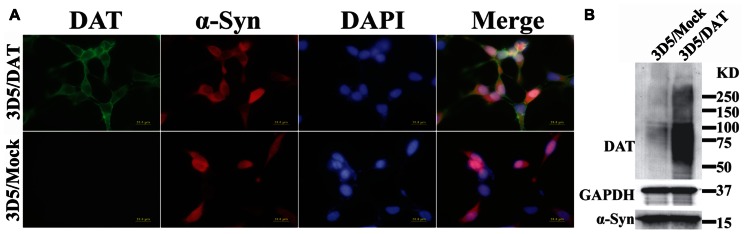
**Expression of dopamine transporter and α-syn in 3D5/DAT cell line.** 3D5 cells with DAT or mock transfection were maintained in 24-well plates containing coverglasses and 6-well plates and used for **(A)** immunocytochemistry and **(B)** western blotting, respectively, after 8 days of RA differentiation and α-syn induction. Dual immunofluorescence labeling demonstrated the expression both DAT (green) and α-syn (red) proteins in transfected cells, whereas mock transfected displayed only α-syn immunoreactivities. Nuclei of cultured cells were labeled by DAPI (blue). Western blotting demonstrated the presence of more DAT in lysates prepared from DAT transfected cells than those from mock transfected counterparts. The levels of α-syn or GAPDH in lysates from the transfected and mock transfected cells are comparable.

To verify that DAT transfected 3D5 cells express DAT, we compared them with mock transfected counterparts by immunocytochemistry (**Figure 1A**) and western blotting (**Figure 1B**). DAT immunoreactivities were readily detected only in the DAT transfected, whereas α-syn immunoreactivities were detected in both DAT and mock transfected cells with Tet-Off induction. Western blotting of cell lysates demonstrated robust signals of DAT expression in the transfected and very weak signals in mock transfected controls. It is worth noting that very low expression of DAT in mock transfectant can be detected by western blotting but not immunocytochemistry might be due to the difference of sensitivity between them.

### TIME-DEPENDENT INCREASES OF LPO IN NON-INDUCED 3D5/DAT CELLS WITH AA TREATMENT

**FIGURE 2:**
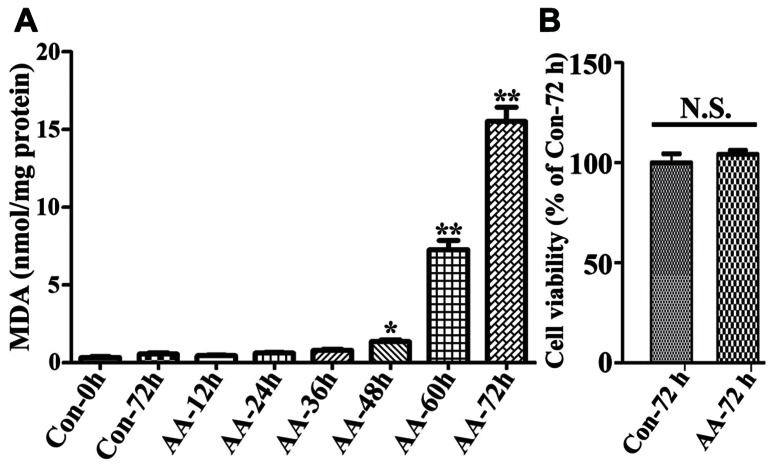
**Time-dependent induction of LPO in RA-differentiated, non-induced 3D5/DAT cells by AA treatment.** 3D5/DAT cells with 8 days of RA-elicited differentiation and without α-syn induction were treated with AA for 12, 24, 36, 48, 60, and 72 h, referred to as AA-12h, AA-24h, AA-36h, AA-48h, AA-60h, and AA-72h, respectively, or without for 0 and 72 h (Con-0h, Con-72h). They were harvested and used for **(A)** LPO assay to determine MDA levels per mg protein. **(B)** Sibling cultures of AA-72h and Con-72h were used for Calcein AM assay to assess cell viability. Bar graphs summarized data derived from three independent experiments using different sets of cultures. The levels of LPO detected in cultures with 48, 60, and 72 h of AA exposure were 3, 19, and 40 times of that in Con72h cultures, and the differences are statistically significant. Error bars represent standard errors of the mean (SEM). (*p < 0.05 and **p < 0.01 comparing to Con-0h, *n* = 3) In contrast to that displayed by LPO assay, there was no significant difference between cultures with and without 72 h of AA exposure in regard to their viability.

To demonstrate the effects of AA on LPO we used non-induced 3D5/DAT cells (i.e., without α-syn overexpression) with 8 days of RA-elicited neuronal differentiation, and treated them with or without AA. In comparison to vehicle-treated cells (regarded as controls), the AA-treated displayed a time-dependent increase of MDA, a product of LPO. Significant differences between the AA-treated and controls were detected only after 48 h or longer durations of treatment (**Figure 2A**). On overage, the amount of MDA detected in cultures with 48, 60, and 72 h of AA exposure was about 4, 19, and 40 times, respectively, of that present in their counterparts without AA exposure for 72 h. Such increase of LPO, however, did not have significant impacts on cell viability upon the AA exposure (**Figure 2B**), indicating that differentiated 3D5/DAT cells without induced α-syn expression can tolerate a 3-day surge of LPO.

### LPO FACILITATES α-syn OLIGOMER ACCUMULATION AND CASPASE 3 ACTIVATION

**FIGURE 3:**
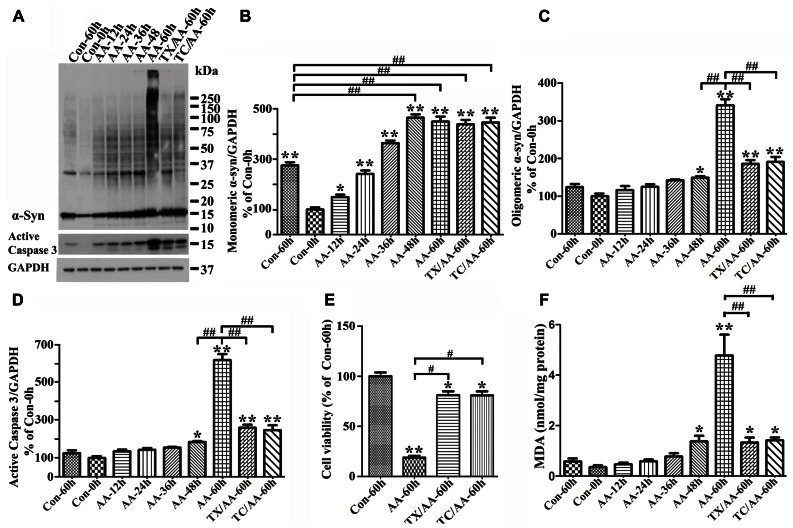
**Facilitation of monomeric and oligomeric α-syn accumulation and caspase activation by LPO.** 3D5/DAT cells with 8 days of RA-elicited differentiation and Tet-Off induced α-syn expression were exposed to AA for 12, 24, 36, 48, and 60 h (regarded as AA-12h, AA-24h, AA-36h, AA-48h, and AA-60h), treated with AA plus TX or AA plus TC in for 60 h (TX/AA-60h and TC/AA-60h) or without any treatment for 0 or 60 h (Con-0h, Con 60h). Cells harvested from each set of culture were split into two and used for western blotting and LPO assay. Sibling cultures of con-60h, AA-60h, TX/AA-60h, and TC/AA-60h were subjected to Calcein assay. **(A)** Western blotting of cell lysates with antibodies to α-syn, active caspase 3 and GAPDH. **(B–D)** Bar graphs represent data obtained from quantitation of immunoreactivities displayed in **(A)** and western blot from two additional experiments. The levels of immunoreactivity detected in controls (Con-0h) are regarded as 100%. Our results showed more α-syn oligomers and active caspase 3 in cultures with 60 h of AA treatment, and such increase was reduced in cultures co-treated with AA plus TX or TC. **(E)** Calcein assay of con-60h, AA-60h, TX/AA-60h, and TC/AA-60h cultures. Results showed that AA-induced death can be blocked significantly by co-treatment with TX or TC. **(F)** LPO assay for all samples, demonstrating a significant increase of LPO levels after 48 h or longer exposure of cultures to AA when compared to controls (compared AA-48 to con-0h). Such changes were reduced significantly by TX or TC co-treatment. The results are representative of three independent experiments. Error bars represent SEM. Number sign(s) marked significant differences between two groups linked by bracket (^#^*p* < 0.05, ^##^*p* < 0.01). Asterisk(s) indicated significant differences between the treated group and Con0h (*p < 0.05, **p < 0.01).

To determine the effects of LPO on α-syn assembly, we used 3D5/DAT cells with induced α-syn expression. After treatment with or without AA for up to 60 h these cells were analyzed by LPO assay and western blotting. Similar to what was observed in 3D5/DAT cells without α-syn induction, significant increases of LPO were detected in samples from cultures that have been exposed to AA for 48 h or longer durations. The extents of LPO increase were comparable between the AA-treated cultures with and without α-syn induction. However, more α-syn accumulation were detected in cultures with AA treatment than without, and more in those with longer durations of AA exposure (**Figure 3A**). Such increase of α-syn accumulation was contributed not only by increasing the level of monomeric α-syn but also that of different sizes oligomeric α-syn species (≥33kDa;**Figures 3A–C**). We noted that cultures with 60 h of AA treatment contained far more large sizes α-syn oligomers (>75 kDa) than those with shorter treatment, and some of the large oligomers were too large to enter the separation gel (**Figures 3A,C**). Moreover, small oligomers of size consistent with that of α-syn dimer (~30 kDa) were less abundant in cultures with 60 h of AA exposure than those with shorter periods of treatment. Such shifting of α-syn gel electrophoretic profiles indicates that the assembly of α-syn in these cultures is AA-elicited and time-dependent. Besides α-syn accumulation, AA exposure also resulted in increase of caspase 3 activation (**Figures 3A,D**) and decrease of the viability of Tet-Off-induced cells (**Figure 3E**) in a time-dependent manner.

To determine whether AA-elicited accumulation of α-syn assembly is linked to LPO, we included cultures co-treated with AA and TX or AA and TC in our studies of Tet-Off-induced 3D5/DAT cells. TX is a potent water-soluble vitamin E analog that has been used in numerous studies to block LPO by scavenging ROS before they attack intracellular lipid (Wolf, 2005), and TC is a potent inhibitor of long-chain fatty acyl CoA synthetase that can effectively inhibit AA uptake and incorporation into cell (Hartman et al., 1989). Our LPO assay showed that TX or TC co-treatment protects cultures from AA-elicited LPO as expected. The level of MDA in such co-treated cultures was about 28% of that in culture with AA treatment only (**Figure 3F**). Importantly, TX or TC co-treatment was highly effective to reduce the effects of AA on accumulation of oligomeric α-syn species, caspase 3 activation and cell death (**Figures 3A–E**).

**FIGURE 4:**
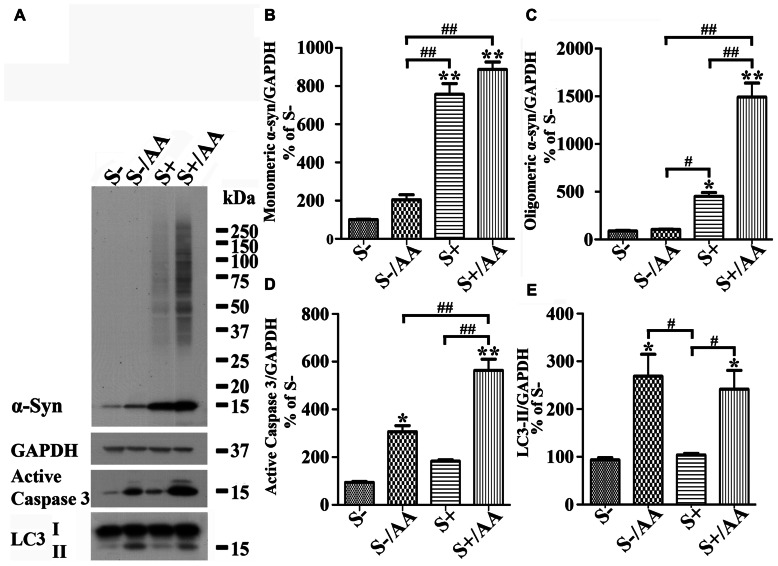
**Differential responses of cells with and without α-syn induction to AA exposure.** 3D5/DAT cells with 8 days of RA-elicited differentiation plus Tet-Off induced α-syn expression and their non-induced counterparts were treated with AA for 60 h and referred to as S+/AA and S-/AA, sibling cultures without treatment were used as controls and referred to as S+ and S-, respectively. **(A)** Western blotting of cell lysates with antibodies to α-syn, LC3, active caspase 3, and GAPDH. **(B–E)** Bar graphs represent data obtained from quantitation of immunoreactivities displayed in **(A)** and western blot from two additional experiments. The results are representative of three independent experiments. Error bars represent SEM. Number sign(s) marked significant differences between two groups linked by bracket (^#^*p* < 0.05, ^##^*p* < 0.01). Asterisk(s) indicated significant differences between the treated group and Con0h (*p < 0.05, **p < 0.01). Our results showed that S+/AA were demonstrated to accumulate much more α-syn than S-/AA. Most of the increase of α-syn observed in S-/AA is monomer, whereas that in S+/AA is oligomers and monomers. The results of immunoblotting also showed that AA exposure heightened the level of LC3-II and active caspase 3 in both S-/AA and S+/AA cultures, but its impacts on caspase 3 was greater for the S+/AA than S-/AA cultures.

Increases of monomeric α-syn and active caspase 3 levels were also detected in non-induced 3D5/DAT cultures with AA exposure (**Figures 4A,D**, compared S- to S-/AA). However, the extent is much smaller than that demonstrated in cells with α-syn induction (**Figures 4A,D**, compared S-/AA to S+/AA). Importantly, oligomeric α-syn was not detected in non-induced cultures with AA exposure and the level of active caspase 3 is higher in their induced counterparts (**Figures 4A,C,D**).

Together, our findings support the notion that LPO plays a role in promoting α-syn accumulation/assembly in Tet-Off-induced cells, that expression of certain levels of α-syn monomers can facilitate the accumulation of large sizes α-syn oligomers and that these events are associated with heightened caspase 3 activation.

### α-syn EXPRESSION/ACCUMULATION INCREASES THE SENSITIVITY OF CELLS TO AA-ELICITED CYTOTOXICITY IN AN α-syn CONCENTRATION-DEPENDENT MANNER

**FIGURE 5:**
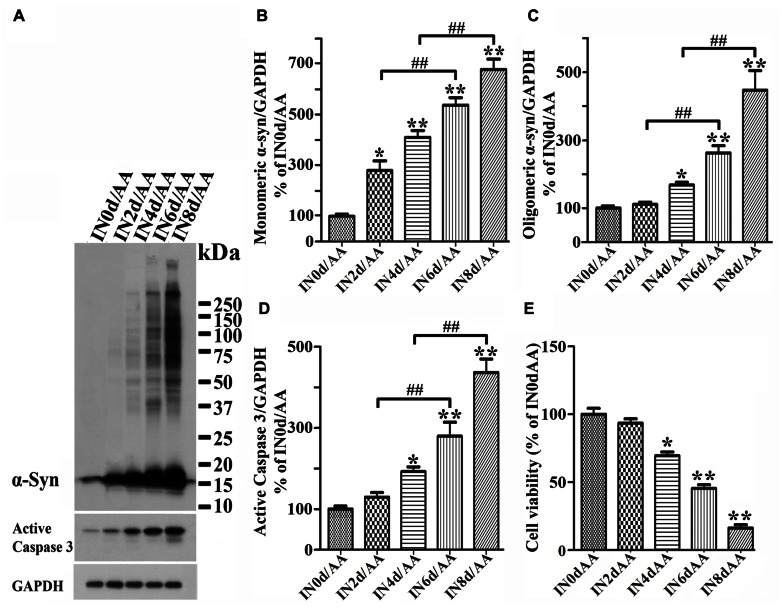
**Induced α-synuclein expression increases the sensitivity of cells to AA-elicited cytotoxicity.** Five sets of differentiated 3D5/DAT cells with 0, 2, 4, 6, and 8 days of induced α-syn expression were exposed to AA for 60 h and referred to as IN0d/AA, IN2d/AA, IN4d/AA, IN6d/AA, and IN8d/AA, respectively. **(A)** Western blotting of lysates derived from these cells with antibodies to α-syn, active caspase 3 as well as GAPDH showed a time-dependent increase of monomeric and oligomeric α-syn species as well as that of active form of caspase 3. **(B–D)** Bar graphs summarized quantitative analysis of α-syn and active capase 3 immunoreactivities displayed in **(A)** and normalized with GAPDH as well in western blot from additional experiments. The average values of control groups (IN0d/AA) were set as 100%. **(E)** Calcein assay showed an inverse relationship between cell viability and the duration of α-syn induction in cultures with AA exposure. Significant decrease of cell viability was detected in cultures with 4 days or longer durations of induced α-syn expression plus AA exposure when compared to non-induced counterparts (IN0d/AA). The results are representative of three independent experiments. Error bars represent SEM. The changes of levels of various proteins or cell viability are statistically significant. (*p < 0.05, **p < 0.01 comparing to INd0/AA; ^#^*p* < 0.05, ^##^*p* < 0.01, comparing subsets linked by bracket).

To determine whether cytotoxicity caused by AA treatment is linked to expression/accumulation of α-syn, we seeded RA-differentiated 3D5/DAT cells in 48-well plate, induced the cultures to express α-syn for 0, 2, 4, 6, or 8 days then exposed them to AA for 60 h to initiate LPO. Cells in half of the plate were harvested for western blotting analysis, and the rest were used for calcein assay cell to evaluate cell viability. Western blotting of cell lysates showed AA-treated cultures with longer durations of Tet-Off induction having more α-syn accumulation and caspase 3 activation than non-treated counterparts (**Figures 5A–D**). In AA-treated cultures, significant increase of monomeric α-syn species was readily detected after 2 days of Tet-Off induction (**Figure 5B**), whereas increase of oligomeric α-syn became apparent after longer durations of induction (**Figure 5C**). Some of such oligomers were greater than 250 kDa in size. Importantly, AA-treated cultures with longer durations of induced α-syn expression displayed lower cell viability (**Figure 5E**), and AA treatment of cultures without the induced α-syn expression had very little effects on cell viability (**Figure 2B**). Such inverse relationship between the length of α-syn induction and the magnitude of AA-elicited toxicity suggests that the sensitivity of cells to insults increases in an α-syn concentration-dependent manner.

### DOPAMINE PROTECTS CELLS FROM TOXIC α-syn ACCUMULATION AND REDUCES LPO ELICITED BY AA TREATMENT

**FIGURE 6:**
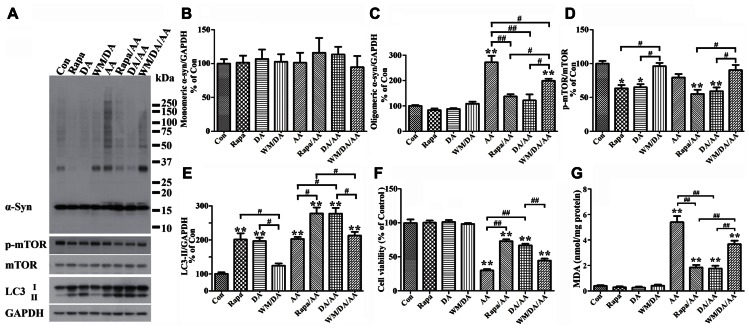
**Protection of cells from AA-elicited α-syn accumulation, LPO, and cytotoxicity by autophagy activation.** Four sets of 3D5/DAT cells with 8 days of differentiation and α-syn induction were exposed for 60 h to AA, AA plus Rapa, AA plus DA, AA plus DA and WM, and regarded as AA, Rapa/AA, DA/AA, and WM/DA/AA, respectively. Additional four sibling cultures were similarly treated but without AA and named as Con, Rapa, DA, and WM/DA. Cells harvested from each subset were split into two parts for western blotting and LPO assay. Duplicate cultures were included for cell viability assessment. **(A)** Western blotting of lysates of all samples with antibodies to α-syn, phosphorylated mTOR (p-mTOR), total mTOR, LC3, and GAPDH. **(B–E)** Bar graphs summarized quantitative analysis of immunoreactivities displayed in **(A)** with GAPDH normalization as well in western blot from additional experiments. Results demonstrated that DA or Rapa treatment decreases significantly the relative amount of p-mTOR to mTOR and increase that of LC3-II to GAPDH, suggesting an activation of autophagy or accumulation of autophagosomes. These treatments also significantly reduce the level of AA-elicited α-syn oligomer accumulation. The effects of DA or Rapa (data not shown) was blocked/reduced by WM co-treatment indicating that autophagy inhibition counters the effects of DA on reducing α-syn accumulation. **(F)** Calcein assay showed that Rapa treatment is as effective as DA in protecting cells from AA-elicited cytotoxicity, and that WM co-treatment reduces the beneficial effects of DA on cell viability. **(G)** LPO assay showed that Rapa treatment is as effective as DA in lowering LPO, and that WM co-treatment can reduce the impact of DA on LPO. (*p < 0.05, **p < 0.01 comparing to Con; ^#^*p* < 0.05, ^##^*p* < 0.01, comparing subsets linked by bracket). Error bars represent SEM.

It has been reported that presence of DA inhibits fibrillization of recombinant α-syn, and that such effect is exerted by oxidative products of DA, which can covalently modify α-syn as well as induce conformational change of α-syn to form SDS-resistant soluble oligomers without having beta sheet structures (Cappai et al., 2005). Based on this scenario, one would expect to find more SDS-resistant α-syn assemblies in cultures with DA exposure than without. To determine whether or not this is the case we used differentiated, Tet-Off-induced 3D5/DAT cells, and treated them with DA, without DA, or with DA plus AA. The concentration of DA used in most of this study was 50 μM, which is the highest concentration regarded as physiological by others in previous studies (Grace and Bunney, 1983; Li et al., 2004; Heien et al., 2005). Contrary to what was expected, cultures with DA exposure contained less α-syn oligomers (about 33 kDa in size) than those without (**Figure 6A**, compared DA to Con). Moreover, cells with DA and AA co-exposure also accumulated significantly less α-syn than those treated with AA alone (**Figures 6A, C**, compared DA/AA to AA), and most of the differences were due to decrease of oligomeric α-syn species (**Figures 6B,C**, compared DA/AA to AA).

In light of our findings regarding a close link between LPO and α-syn self-interactions, we investigated whether DA treatment can affect LPO levels. As shown in **Figure 6G**, samples from cultures with DA plus AA treatment contained lower levels of MDA than those treated with AA only, and the differences were statistically significant. DA treatment of cells without AA exposure also caused some decrease of MDA levels when compared to non-treated counterparts. However, this change is statistically insignificant presumably because the extent of LPO was very low to start with.

We next investigated whether reduction of α-syn accumulation and LPO by DA treatment has any impacts on cell viability, since it has been suggested that soluble oligomers may be cytotoxic. By calcein assay we showed that DA exposure significantly protects cultures from the cytotoxic effects of AA treatment (**Figure 6F**). Together our findings indicate that presence of DA can protect cells from accumulation of oligomeric α-syn, elevation of LPO and cell death.

### DA TREATMENT PROTECTS CELLS FROM AA-ELICITED α-syn ACCUMULATION, LPO, AND CYTOTOXICITY VIA AUTOPHAGY ACTIVATION

#### α-Syn accumulation

To determine how DA prevents cells from AA-elicited α-syn accumulation, we tested whether autophagy plays a role, since it has been reported that DA treatment can lead to increase of LC3-II (Gimenez-Xavier et al., 2009). Western blotting of lysates of Tet-Off-induced, differentiated 3D5/DAT cultures with antibodies to proteins relevant to autophagy showed that DA treatment causes increase of the ratio of LC3-II/GAPDH (**Figure 6E**) and decrease the ratio of phosphorylated mTOR (p-mTOR)/total mTOR levels, suggesting DA may function as an autophagy inducer (**Figures 6A,D,E**, compared DA to Con).

Our studies of cells co-treated with DA plus AA as well as those treated with AA alone also showed stronger signals of LC3-II activation, lower ratio of p-mTOR/total mTOR levels and less α-syn accumulation in the co-treated, suggesting autophagy activation may be a mechanism used by DA to prevent cells from AA-elicited α-syn accumulation (**Figures 6A,D,E**, compared lanes DA/AA and AA). The role of autophagy in reducing α-syn accumulation was supported further by data obtained from studying AA-treated cultures co-treated with Rapa, which is an inducer of autophagy. Cultures with Rapa and AA co-treatment accumulated less α-syn oligomers than those treated with AA (**Figures 6A,C**, compared Rapa/AA to AA).

To further verify the role of autophagy in reducing α-syn accumulation we compared cultures treated with DA to those co-treated with DA plus AA or co-treated with DA, AA plus WM (an autophagy inhibitor). Cultures with WM co-treatment were found to accumulate more α-syn and p-mTOR/mTOR (**Figures 6A,D**, compare DA to WM/DA or DA/AA to WM/DA/AA), but have lower LC3-II/GAPDH ratio than those without the co-treatment (**Figure 6E**). Together our data supports a role of autophagy in reducing α-syn accumulation in DA-treated cultures.

#### Lipid peroxidation and cell viability

Besides α-syn accumulation, we have tested whether DA or Rapa treatment can protect cells from the raise of LPO or decrease of cell survival caused by AA exposure. We demonstrated that AA-elicited LPO can be significantly reduced by Rapa treatment (**Figure 6G**, compared Rapa/AA to AA), and the extent of reduction is comparable to that observed in cells co-treated with DA plus AA. Such beneficial effects of DA on LPO were significantly blocked by WM treatment, albeit partially (**Figure 6G**, compared WM/DA/AA to DA/AA). As to cell viability, Rapa was as effective as DA in reducing the detrimental effects of AA on cell survival (**Figure 6F**, compared DA/AA to AA). In comparison, cultures treated with WM plus DA and AA were less viable than those treated with DA plus AA. Together, these findings support a role of autophagy in reducing the cytotoxicity elicited by AA treatment.

### AA TREATMENT-INDUCED ACCUMULATION OF LC3-II AND LYSOSOMAL DYSFUNCTION

It is worth noting that cells treated with AA, regardless of whether they have α-syn induction or not, have a significantly higher LC3-II/GAPDH ratio than non-treated control. We speculated that the changes of LC3-II/GAPDH ratio may reflect decrease of autophagy flux via disturbance of lysosomes, since the treated cells have more α-syn accumulation (i.e., monomers in non-induced and monomers plus oligomers in induced), and α-syn can be degraded by lysosomal proteases (Lee et al., 2004; Sevlever et al., 2008; Mak et al., 2010). Moreover, studies of lysosomes isolated from liver have demonstrated that AA treatment can alter lysosomal permeability to both potassium ions and protons and enhance the lysosomal osmotic sensitivity (Zhang et al., 2006).

**FIGURE 7:**
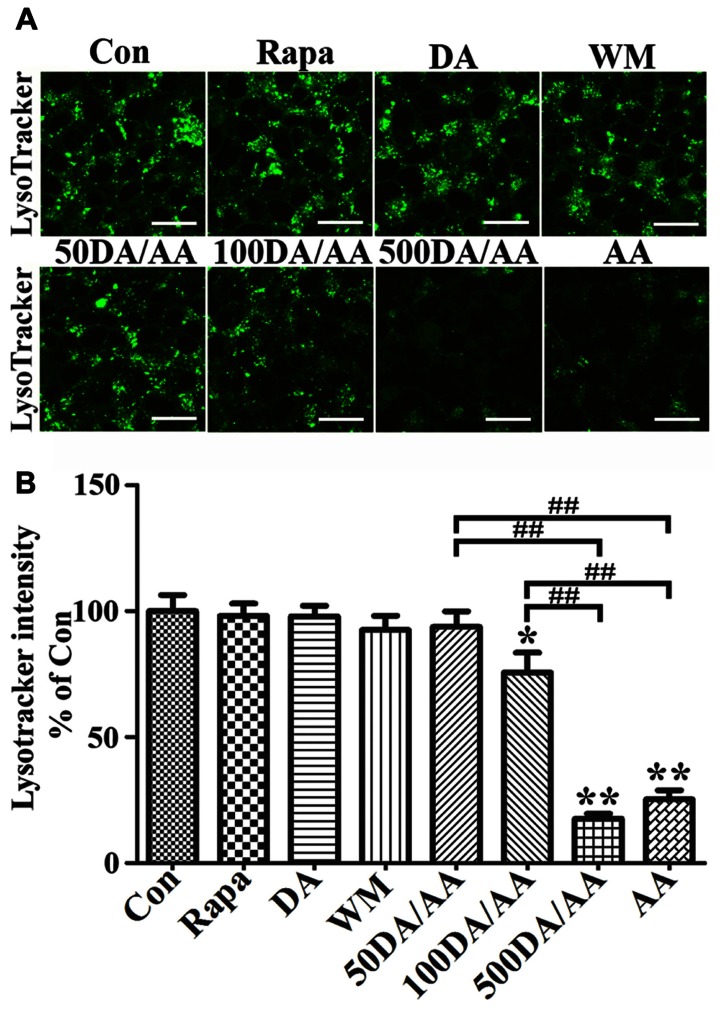
**AA treatment induces lysosome dysfunction in 3D5/DAT cells.** Eight sets of 3D5/DAT cells cultured on Lab-Tek Chambered Coverglass were subjected to 8 days of differentiation and α-syn induction. They were treated for 60 h with Rapa, DA (50, 100, or 500 μM), WM, AA, or without any treatment to serve as control (Con). At the end of treatment, LysoTracker Green (invitrogen) was added at a final concentration of 100 nM to each well for 1 h. Cells were then evaluated for lysosomal function by confocal microscopy. **(A)** Representative images of LysoTracker labeled cells in different groups were shown. **(B)** Bar graph summarized data from quantitative and statistical analyses of more than 20 images captured from each group. (*p < 0.05, **p < 0.01 comparing to Con; ^##^*p* < 0.01, comparing subsets linked by bracket).

To study the effects of AA on lysosomes, we incubated 3D5/DAT cells with LysoTracker Green DND-26, which is a fluorescence agent that stains acidic compartments in live cells (**Figure 7A**). We compared the intensities of fluorescence signals emitted from cells treated with AA to those without. Cells with α-syn induction and treated with AA for 60 h were found to display significantly weaker fluorescence signals than those without (Con) or treated with only Rapa, WM or DA at 50 μM (**Figures 7A,B**) and 500 μM (data not shown). The results support that AA treatment causes an increase of lysosomal membrane permeability, and that such change is probably responsible for increase of α-syn accumulation in α-syn-induced cells. Similar results were obtained from LysoTracker labeling of non-induced cells (data not shown). In order to further understand how treatment with DA at physiological concentration protects cells from AA-elicited changes, we compared cells with DA and AA co-treatment (50DA/AA) and those with AA treatment alone (AA) for LysoTracker staining. Cultures with 50DA/AA were found to display stronger florescence signals than AA (**Figures 7A,B**, 50DA/AA versus AA). The differences are statistically significant. Importantly, the co-treated were comparable to untreated controls as to fluorescence signal intensity (**Figure 7**, 50DA/AA versus Con), indicating prevention of lysosomal damages is a mean for protection of AA-elicited damages. LysoTracker staining showed a significant decrease of fluorescence signals in cell treated with AA plus ≥100 μM when compared to controls or those treated with AA plus 50 μM DA (**Figures 7A,B**).

### BIPHASIC EFFECTS OF DA EXPOSURE ON α-syn OLIGOMER ACCUMULATION AND CELL SURVIVAL

**FIGURE 8:**
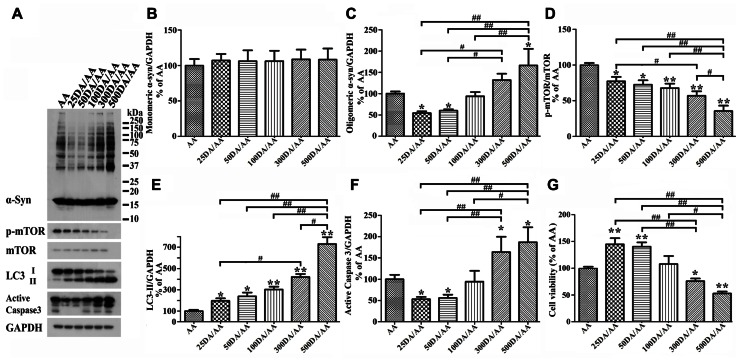
**Biphasic effects of DA exposure on α-syn oligomer accumulation and cell survival.** 3D5/DAT cells seeded on 48-well plates with 8 days of differentiation and α-syn induction were exposed to AA plus 0, 25, 50, 100, 300, or 500 μM DA for 60 h and referred to as AA, 25DA/AA, 50DA/AA, 100DA/AA, 300DA/AA, 500DA/AA, respectively. They were used for western blotting with antibodies raised to α-syn, p-mTOR, mTOR, LC3, active caspase 3, and GAPDH and for calcein assay. **(A)** Western blotting of cell lysates with antibodies to α-syn, p-mTOR, mTOR, LC3, active caspase 3, and GAPDH. **(B–F)** Bar graphs summarized data from quantitation of the immunoreactivities of various proteins labeled in **(A)** with GAPDH normalization and that from additional experiments. Results showed that co-treatment of cultures with 25 or 50 μM DA plus AA (regarded as 25DA/AA, and 50DA/AA) caused decrease of both oligomeric α-syn accumulation and active caspase 3 levels, but similar co-treatment with 300 or 500 μM DA plus AA led to increase of these parameters above the levels demonstrated in cells with AA exposure only. By contrast, the relative level of autophagy markers (i.e., p-mTOR/mTOR and LC3-II/GAPDH) changed in a DA concentration-dependent manner. **(G)** DA at 50 μM or lower concentrations significantly protected cultures from the detrimental impacts of AA exposure on cell viability, whereas at higher concentrations (>300 μM) the presence of DA caused a significant reduction of cell viability (*p < 0.05, **p < 0.01 comparing to AA; #*p* < 0.05, ##*p* < 0.01, comparing subsets linked by bracket).

It has been reported that autophagy pathway is capable of killing cells (Jaeger and Wyss-Coray, 2009; Choubey et al., 2011). To determine whether DA at concentration higher than 50 μM can still protect Tet-Off-induced 3D5/DAT cultures from AA-elicited toxicity and α-syn accumulation, we compared cultures treated for 60 h with AA plus DA at different concentrations, ranging from 0 to 500 μM. Western blotting of lysates from these cells showed accumulation of lower levels of α-syn in those co-treated with AA plus 25 or 50 μM DA than cultures treated with AA only (i.e., AA, **Figures 8A,B** and **C**). By contrast cultures co-treated with AA and DA at 300 μM or higher concentrations accumulated more α-syn than those treated with AA alone. Such biphasic effects of DA did not apply to autophagy markers as the ratio of p-mTOR/mTOR and LC3-II/GAPDH changed in a DA concentration-dependent manner (**Figures 8A,D,E**). Cultures treated with AA and DA at 300 μM or higher concentrations contained more active caspase 3 and were less viable than those with AA treatment only (**Figures 8A,F,G**, compared 300DA/AA to 50DA/AA), while those treated with AA and DA at lower than 300 μM showed less caspase 3 activation than the AA-treated. Thus, depending on its concentration, the presence of DA can have opposite effects on neuronal cell survival and α-syn accumulation. This is consistent with previous reports that autophagy pathway can be involved in both survival and death of cells (Cherra and Chu, 2008; Jaeger and Wyss-Coray, 2009).

Next, we examined cultures exposed to AA plus DA at different concentrations upon LysoTracker labeling (**Figures 7A,B**). In contrast to what was demonstrated in 50 μM DA plus AA-treated cells, those co-treated with DA at higher concentrations displayed significantly weaker fluorescent signals than controls. Fluoresce signals displayed by cultures co-treated with 50 or 100 μM DA and AA are significantly more intense than those detected in those co-treated with 500 μM DA and AA. Moreover, the 500 μM DA co-treated tend to show weaker lysosomal staining than those treated with AA only, although statistically insignificant(**Figures 7A,B**).

## DISCUSSIONS

Information accumulated from numerous studies suggests that PD is associated with perturbation of multiple pathways to result in abnormal energy metabolism, oxidative and ER stresses, increase of α-syn aggregation, synaptic dysfunction, and other damages (Jenner, 1991; Fahn and Cohen, 1992; Simon and Beal, 2002; Dauer and Przedborski, 2003; Picconi et al., 2012). Many of the changes were detected in brain tissue of subjects at early stages of PD (Braak et al., 2003). Although it remains uncertain if a certain pathway was affected before others, it is reasonably to consider that abnormalities displayed at different stages of PD likely reflect the cross-talks among different pathways. Oxidative stress has been placed as a key component in PD pathogenesis (Jenner, 1991; Fahn and Cohen, 1992; Tieu et al., 2003), since it is known to link ROS production to a variety of metabolic outcomes, including oxidative modification of proteins and lipids (Danielson and Andersen, 2008; Radak et al., 2011), which in brain tissue are enriched in PUFAs (Rapoport, 2008; Whelan, 2008). Polyunsaturated acyl chains of phospholipids or PUFAs such as AA, docosahexaenoic acid, and linoleic acid are highly susceptible to peroxidation and breakdown to form a variety of lipid-derived aldehydes and ketones (Frankel, 1984). In this regard, it has been reported that at very early stages of PD (stages 1 and 2), before the appearance of α-syn aggregates, neurons in substantia nigra display signs of oxidative damage to lipid, and this brain region at stage 3 displays punctate aggregates containing α-syn modified by oxidization among other types of posttranslational modifications (Ferrer et al., 2011). *In vitro* studies have shown that modification of α-syn by LPO products (e.g., 4-hydroxynonenal) leads to**inhibition of α-syn fibrillization and formation of soluble oligomers (Qin et al., 2007). Addition of 4-hydroxynonenal modified α-syn oligomers to neuronal cell cultures caused cell death, suggesting the oligomers are cytotoxic (Qin et al., 2007). It has long been considered that a significant contributing factor rendering dopaminergic neurons more susceptible to degeneration than other neurons is the high susceptibility of DA to undergo autoxidation (Miyazaki and Asanuma, 2008). Cell-based studies have shown that increase the steady-state levels of DA can inhibit formation of α-syn aggregates and induce the formation of innocuous oligomers (Yamakawa et al., 2010) and exposure to DA at non-physiological concentrations caused cell death (Gomez-Santos et al., 2003; Benchoua et al., 2008).

To understand further the role of lipid oxidation, DA and α-syn and their interactions in PD, we performed a series of studies using cultured cells of human origin generated in present studies. The cells, regarded as 3D5/DAT, are inducible to express human wild-type α-syn and express DAT stably. They displayed neuronal phenotype in response to RA treatment (Ko et al., 2008). We employed AA exposure as a mean to promote LPO in these cells and have demonstrated that the magnitude of LPO is AA exposure time- (**Figures 2** and **3**) and concentration-dependent as reported previously (Canuto et al., 1991; Schonberg et al., 2006). In our cultures, a minimum 2 days of exposure to 250 μM AA was required to cause a significant elevation of LPO levels (**Figure 2A**), and longer durations of exposure were required to achieve the same effect if lower AA concentrations were used (data not shown). This is due to the fact that LPO is a free radical-mediated chain reaction, involving initiation, propagation, and termination (Young and McEneny, 2001; Farooqui and Farooqui, 2011) and that products of LPO are highly reactive to make covalent modifications of macromolecules (Grimsrud et al., 2008; Gueraud et al., 2010).

Although it has been reported that overexpression of α-syn can increase intracellular ROS levels (Junn and Mouradian, 2002) in other cell types, our previous studies have shown that the level of ROS in 3D5 cells is not significantly affected by α-syn overexpression (Takahashi et al., 2007). In fact, during the course of AA treatment (i.e., up to 72 h), cells with α-syn induction displayed LPO at the extent comparable to that detected in corresponding samples without the induction. Therefore, that α-syn overexpression has no influence on LPO in present study. We also demonstrated that AA exposure can cause caspase 3 activation as reported previously (Vento et al., 2000; Saitoh et al., 2003). The increase of active caspase 3 by AA treatment is time-dependent and the pattern of changes parallels that of AA-elicited LPO, showing marked and sustained elevation in cultures treated for 60 h or longer. Moreover, cells with α-syn induction contained more active caspase 3 and exhibited less viability than their non-induced counterparts. The changes of caspase activation and cell viability probably are linked to LPO, since they were significantly reduced in cells co-treated either with AA plus LPO scavenger (TX) or AA plus its uptake inhibitor (TC; **Figure 3**). It is worth noting that cells expressing more α-syn are more responsive to activation of caspase 3 and to decrease of viability elicited by AA exposure, which is consistent with previous studies that α-syn overexpression increases the sensitivity of neuronal cells to different insults (Orth et al., 2003; Nieto et al., 2006; Bisaglia et al., 2010; Jiang et al., 2010,^2013^). However, the mechanism underlying that α-syn modulates the level of active caspase 3 in AA-treated cultures is unclear. Since the increase of active caspase 3 is concomitant with that of LPO and oligomeric α-syn, it is possible that in present study LPO induced α-syn oligomers have α-syn dose-dependent toxicity, thus trigger the activation of caspase 3 in a stronger manner than LPO alone, and render cells bearing more α-syn more vulnerable to LPO insults. The exact mechanism remains further investigations.

Another consequence of AA exposure to cells with α-syn induction is enhanced accumulation of α-syn monomers and different sizes oligomers. Such responses were also detected in cells without the induction, but the magnitude is much smaller and more apparent for monomers (**Figure 4**). The AA-elicited α-syn oligomers are detectable by SDS-PAGE, hence are deemed as SDS-resistant. Accumulation of these oligomers are results of either acceleration of α-syn assembly and/or decrease of its degradation. The latter possibility was tested by examining the levels of LC3-II/GAPDH, p-mTOR versus total mTOR, and the integrity of lysosome membranes. Although western blot studies showed that AA treatment affects the ratio of LC3-II/GAPDH and that of p-mTOR/total mTOR, these changes do not guarantee autophagic degradation, since protein degradation via autophagy-lysosome system requires fusion of autophagosomes to functional lysosomes. LysoTracker labeling of 3D5/DAT cells demonstrated that the integrity of lysosomes was compromised by AA treatment (**Figure 7**). Therefore, AA-elicited α-syn accumulation is a result of decreased protein degradation and changes of autophagy markers detected in the AA-treated cells reflects likely the inhibition of autophagic degradation of LC3-II (i.e., accumulation of autophagosomes).

We have demonstrated that addition of DA at physiological concentrations to AA-treated 3D5/DAT cells leads to not only a marked decrease of oligomeric α-syn, decrease of LPO and active caspase 3, but also increase of cell viability. Opposite results were obtained from similar analysis of cells with exposure to DA at higher concentration. Together the results indicate that DA treatment has biphasic effects; pro and against cell survival at low and high concentrations, respectively. How DA exerts the opposite effects observed in our studies is an issue of interest. We explored the possibility that at low concentrations DA treatment may activate autophagy to degrade α-syn and other proteins susceptible to modification by LPO products, some which can be cytotoxic (Camandola et al., 2000). This is supported by data from studies of cells with or without co-treatment with Rapa or WM (**Figure 6**). Rapa exposure has the same impacts as low DA on blocking the changes elicited by AA, whereas WM exposure countered the impacts of DA. Similar to what was demonstrated in the AA-treated, cells with exposure to DA alone or DA plus AA have a higher ratio of LC3-II/GAPDH and lower ratio of p-mTOR/total mTOR than controls. However, it was revealed by LysoTracker labeling that those treated with 50 μM DA, or 50 μM DA plus AA are comparable to untreated controls or those treated with Rapa or WM in respect to lysosome integrity, suggesting that at low concentration DA can protect cells from AA-induced cytotoxicity by maintaining normal lysosomal functions. It is worth noting that increase the steady-state levels of DA via overexpression of tyrosine hydroxylase has been shown to inhibit formation of α-syn aggregates in SH-SY5Y cells overexpressing A53T mutant or wild-type α-syn without causing additional cell death (Mazzulli et al., 2006).

A previous study had exposed rat primary embryonic striatal neurons to different concentration of DA (100, 200, 300, 400, 500 μM) and found that only those exposing to more than 200 μM DA showed loss of cell viability (Benchoua et al., 2008). Later, another study showed that exposure of SH-SY5Y cells to 100 or 500 μM DA resulted in a DA dose-dependent increase of LC3-II, and cell death occurred only in 500 μM DA (Gimenez-Xavier et al., 2009). Consistent with those studies, our results also showed DA dose-dependent increase of LC3-II and that only high concentrations of DA (>300 μM) cause decrease of viability in cells co-exposed to AA. The association between LC3-II increase, protein clearance and cell apoptosis is an issue of interest. As we discussed earlier, and reported previously by others (Mizushima and Yoshimori, 2007), increase of LC3-II/LC3-I alone are not sufficient to support whether autophagy activation leading to successful clearance of proteins/subcellular organelles has occurred or not. Based on our LysoTracker labeling, DA at high concentrations could not protect cells from AA-elicited damages of lysosomal membranes but exacerbate the damage to some extent. Thus, it is reasonable to consider that enhanced α-syn accumulation and reduced cell viability observed in our cultures exposing to high concentration of DA plus AA are due to further blockage of lysosomal degradation of α-syn and other proteins. However, we noted that cells treated with high concentrations of DA alone were comparable to those treated with low DA with respect to LysoTracker labeling intensity and cathepsin D activities (unpublished data). Therefore, additional mechanisms are likely involved in caspase activation/cell death heightened by treatment of cells to AA plus high concentration of DA.

Exposure to DA at high concentrations has previously been reported to cause (i) impairment of proteasomes, (ii) increase in hypoxia-inducible factor 1 alpha, (HIF-1) (iii) accumulation of ubiquitinated proteins and proteins regulated by HIF-1 alpha and involved in apoptosis and/or autophagy (e.g., p53, Puma and Bnip3; Keller et al., 2000; Gomez-Santos et al., 2003; Benchoua et al., 2008; Gimenez-Xavier et al., 2009). Abnormal protein accumulation, in turn, can affect cell survival via activation of caspases to result in apoptotic/autophagic cell death (Qiu et al., 2000; Gomez-Santos et al., 2003). This is supported by our findings of increased levels of active caspase 3 cells treated with high concentrations of DA and decrease of this form of caspase in those treated with low concentrations of DA. We do not know what mechanisms are involved to maintain lysosomal membrane integrity upon exposure to low concentrations of DA. This issue will be pursued in future investigation.

Besides preserving lysosome membrane integrity, low concentration of DA may protect AA-elicited cytotoxicity via other mechanisms. In this regard it has been reported that binding of agonists to D2/D3 receptors can lead to induction of bcl2 and suppression of cytochrome c release from mitochondria (Kihara et al., 2002; Park et al., 2011). However, in our cell model, the possibility of this pathway playing a significant role in protecting cells from AA toxicity by low concentration of DA is low. This is in view of the results obtained from our recent studies, in which an additional transfectant derived from 3D5 cells (i.e., 3D5 cells transfected with pVgRXR and pIND-DAT sequentially) was used. Expression of α-syn and DAT in this new transfectant is inducible by removing tetracycline and adding muristerone, respectively. This transfectant differs from 3D5/DAT, which expresses α-syn and DAT with and without induction, respectively. We found that upon induction of α-syn and DAT expression this new transfectant responded to AA and low concentration of DA in a manner comparable to that of 3D5/DAT cells. We also found that in the absence of induced DAT expression the presence of low DA offered no protective effects on AA toxicity. These findings suggest that DA uptake via DAT rather than its binding to D2/D3 receptors is critical for DA’s protective effects.

Regardless, our results support the possibility that physiological levels of DA has role in maintaining autophagy-lysosomal function. It is worth noting that Rapa has been reported to protect against neuronal death in PD animal models generated from treatment with neurotoxins such MPTP (1-methyl-4-phenyl-1,2,3,6-tetrahydropyridine) and 6-OHDA (6-hydroxydopamine), and to preventL-DOPA (L-3,4-dihydroxyphenylalanine)-induced dyskinesia in 6-OHDA lesioned animals (Malagelada et al., 2010; Feyder et al., 2011). Together with the findings from our cell-based studies, highlight the potential of mTOR signaling cascade as a promising target for development of PD therapeutics.

## Conflict of Interest Statement

The authors declare that the research was conducted in the absence of any commercial or financial relationships that could be construed as a potential conflict of interest.
